# Tackling Energy Loss in Organic Solar Cells via Volatile Solid Additive Strategy

**DOI:** 10.1002/advs.202401330

**Published:** 2024-04-18

**Authors:** Huimin Xiang, Fengbo Sun, Xufan Zheng, Bowen Gao, Panpan Zhu, Tingting Cong, Yuda Li, Xunchang Wang, Renqiang Yang

**Affiliations:** ^1^ Key Laboratory of Optoelectronic Chemical Materials and Devices (Ministry of Education) School of Optoelectronic Materials and Technology Jianghan University Wuhan 430056 China; ^2^ Key Laboratory for Green Process of Ministry of Education School of Chemical Engineering and Pharmacy Wuhan Institute of Technology Wuhan 430205 China

**Keywords:** 1, 4‐bis(iodomethyl)cyclohexane, energy loss, open‐circuit voltage, organic solar cells, volatile solid additive

## Abstract

The energy loss induced open‐circuit voltage (*V_OC_)* deficit hampers the rapid development of state‐of‐the‐art organic solar cells (OSCs), therefore, it is extremely urgent to explore effective strategies to address this issue. Herein, a new volatile solid additive 1,4‐bis(iodomethyl)cyclohexane (DIMCH) featured with concentrated electrostatic potential distribution is utilized to act as a morphology‐directing guest to reduce energy loss in multiple state‐of‐art blend system, leading to one of highest efficiency (18.8%) at the forefront of reported binary OSCs. Volatile DIMCH decreases radiative/non‐radiative recombination induced energy loss (Δ*E*_2_/Δ*E*_3_) by rationally balancing the crystallinity of donors and acceptors and realizing homogeneous network structure of crystal domain with reduced D–A phase separation during the film formation process and weakens energy disorder and trap density in OSCs. It is believed that this study brings not only a profound understanding of emerging volatile solid additives but also a new hope to further reduce energy loss and improve the performance of OSCs.

## Graphical abstract

The greatly reduced energy loss assisted by volatile solid additive 1,4‐bis(iodomethyl)cyclohexane (DIMCH) is demonstrated, and the role of DIMCH in weakening the disparity of imbalanced crystallinity of donor and acceptor, and reducing the energy disorder and trap density is unveiled, and its function to achieve forefront power conversion efficiency of 18.8% for binary organic solar cells.

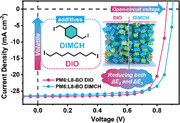

## 1 Introduction

In recent years, the efficiency of organic solar cells (OSCs) has been rapidly developed, reaching more than 19%, which benefits from the development of Y‐series non‐fullerene acceptors (NFAs) and the optimization of device engineering.^[^ ^1^, ^2^, ^3^, ^4^, ^5^, ^6^, ^7^, ^8^, ^9^, ^10^, ^11^, ^12^, ^13^, ^14^ ^]^ Nowadays, in addition to the open‐circuit voltage (*V_OC_*), photovoltaic parameters of OSCs, such as the short‐circuit current density (*J_SC_*) and fill factor (FF) have surpassed more than 80% and 90% of the Shockley–Queisser (SQ) limit, respectively.^[^ ^15^, ^16^, ^17^ ^]^ The large *V_OC_* loss is currently the main obstacle for pursuing the highly efficient OSCs. The *V_OC_* loss can be quantified by the energy loss (*E*_loss_), which typically consists of three components: Δ*E*_1_, Δ*E*_2_, and Δ*E*_3_. Δ*E*_1_ and Δ*E*_2_ represent the radiative recombination losses above and below the bandgap, respectively, while Δ*E_3_* accounts for the non‐radiative recombination loss.^[^ ^18^, ^19^, ^20^ ^]^ The mitigation of *E*_loss_ primarily relies on the reduction of Δ*E*_2_ and Δ*E*_3_. The Δ*E*_2_ can be decreased by reducing the degree of energetic disorder or reorganization energy of active materials,^[^ ^17^, ^20^, ^21^, ^22^ ^]^ while inhibiting non‐radiative recombination can be achieved by reducing the crystallinity difference of donor and acceptor in the active layer, resulting in an efficient decrease in Δ*E*_3_.^[^ ^18^, ^23^, ^24^, ^25^ ^]^ Modifying the chemical structure of photovoltaic materials is one way to reduce Δ*E*_2_ or Δ*E*_3_, but such modifications may lead to more complex and laborious synthesis processes, as well as potentially adverse effects on the morphology of the active layer.^[^ ^26^, ^27^, ^28^ ^]^ As such, it is crucial to establish a simple and effective approach to mitigate both Δ*E*_2_ and Δ*E*_3_, particularly if such a strategy does not inadvertently impair the morphology of the active layer.

Introducing additives has been proven as a simple and effective strategy to optimize molecular crystalline, film fibrillar networks, and even vertical distributions of light‐harvesting components in OSCs. However, the utilization of solvent additives such as 1,8‐diiodooctane (DIO) yields a reduction in *V_OC_* in most instances, probably as a result of strong aggregation of the acceptor molecules within the mixed amorphous region.^[^ ^29^, ^30^, ^31^ ^]^ This phenomenon may give rise to a burst emission, thereby diminishing EQE_EL_ and promoting an increase in non‐radiative recombination loss.^[^ ^32^, ^33^, ^34^ ^]^ It is also important to note that since solvent additives usually have a high boiling point, their residues in active materials can cause energetic disorder in the edge states, leading to extra recombination *E*_loss_.

**Figure 1:**
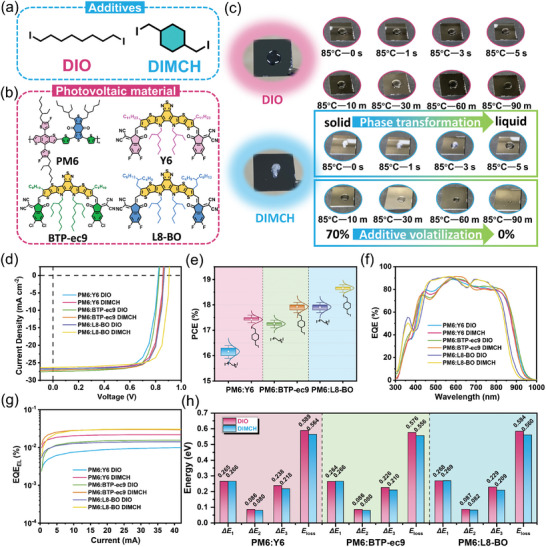
a) Chemical structures of the additives DIO and DIMCH, and photoactive materials. b) Actual plot of DIO and DIMCH over time at 85 °C. c) *J*–*V* curves of OSCs. d) The PCE statistics of OSCs. f) EQE plots of OSCs. e) EQE_EL_ curves of the optimal devices. f) Comparison of Δ*E*_1_, Δ*E*_2_, and Δ*E*_3_ values and *E*_loss_ of OSCs. g) EQE_EL_ curves of the optimal devices. h) Comparison of Δ*E*_1_, Δ*E*_2_, and Δ*E*_3_ values and *E*_loss_ of OSCs.

In comparison to solvent additive with high boiling point, the volatility of solid additive theoretically can avoid the increase of Δ*E*_2_ due to additive residues.^[^ ^7^, ^35^, ^36^, ^37^, ^38^, ^39^ ^]^ Solid additives with high volatility, such as phenoxathiine, octafluoronaphthalene, benzothiadiazole, and their fluorinated analogs, when added to a mixed solution, have the ability to manipulate the formation time of the active layer from the solution state and regulate the timing of acceptor and donor aggregation. Additionally, the using of volatile solid additives can prevent the constant presence of additive residue, which can maintain the ideal morphology, and ultimately the excellent performance and reproducibility of OSCs.^[^ ^40^, ^41^, ^42^, ^43^ ^]^ However, few reports studied new volatile solid additives at molecular level, especially for effectively reducing both Δ*E*_2_ and Δ*E*_3_.^[^ ^11^, ^36^, ^44^, ^45^ ^]^ Herein, the solid additive DIMCH was synthesized by changing the long‐chain alkyl group of the DIO molecule to a more conformationally stable cyclohexyl group (**Figure** **1**a), and to investigate the influence of this conformational change on devices performance. The experimental results revealed that incorporating DIMCH into photovoltaic molecules led to a more ordered molecular stacking and decreased excessive aggregation of the NFA, which, in turn, successfully suppressed emission quenching and ultimately resulted in an improved EQE_EL_. Moreover, the highly volatile property of DIMCH prevented the residue of additives, thereby enabling a further reduction in energy disorder. Compared to devices processed with DIO, the OSCs treated with DIMCH demonstrated superior performance and reduced energy loss (≈0.02–0.03 eV) in various efficient OSCs systems (PM6:Y6; PM6:BTP‐ec9; PM6:L8‐BO). *V_OC_* and fill factor (FF) were significantly enhanced, particularly in the case of PM6:L8‐BO‐based OSCs, which achieved an impressive *V_OC_* of 0.906 V and a power conversion efficiency (PCE) of 18.8%. In conclusion, our results highlight the great potential of such a novel additive in increasing the voltage to build OSCs with better photovoltaic performance.

## 2 Results and Discussion

The chemical structure of DIO and DIMCH is presented in Figure 1a, with additional supporting data containing the synthesis procedures and ^1^H NMR data for DIMCH (Figures S1 and S2, Supporting Information). From the differential scanning calorimetry (DSC) curve (Figure S3, Supporting Information), it can be found that the melting point (*T*_m_) and crystallization temperature (*T*_c_) of DIMCH were 76.9 and 53.0 °C, respectively, which were both lower than the commonly employed thermal annealing temperatures of OSCs (≈85 °C), signifying that DIMCH can influence the morphology of the active layer during the film‐forming stage of spin coating and can also be fine‐tuned during the thermal annealing stage through the phase transitions. In addition, a more intuitive way was utilized to study the volatilization properties of the two additives. As shown in Figure 1c, DIO and DIMCH were placed on a silicon wafer and kept at 85 °C for a certain time to track their states. It can be clearly observed that DIMCH underwent a phase transition from a solid to a liquid within 5 s and completely volatilized after 90 min. While after the same time of heating, there was a large amount of liquid residue of DIO on silicon wafer. These results indicated that DIMCH has a variety of ways to regulate the morphology of the active layer and volatilize in time to avoid residues.

In order to investigate the impact and universality of additives on the photovoltaic performance, three types of OSCs (PM6:Y6, PM6: BTP‐ec9, and PM6:L8‐BO) were fabricated as documented in the attached supplementary information. Representative *J*–*V* curves of different cells were depicted in Figure 1d, which distinctly showed elevated *V_OC_* values after DIMCH treatment. As seen in Figure 1e, the efficiency of OSCs devices with DIMCH in each system was significantly enhanced compared to DIO. As summarized in **Table** **1**, the optimal device efficiencies of PM6:Y6, PM6:BTP‐ec9, and PM6:L8‐BO based OSCs with the inclusion of DIO stood at 16.4%, 17.4%, and 18.1%, respectively, which were comparable to the previously reported performance. In contrast, devices with DIMCH exhibited significantly higher PCE values of 17.6%, 18.1%, and 18.8%, respectively. External quantum efficiency (EQE) spectra and the calculated *J_SC_* values of the optimized devices are presented in Figure 1f, which matched well with the *J_SC_* values measured from *J*–*V* curves. Notably, PM6:L8‐BO based devices exhibited a *J_SC_* of 26.35 mA cm^−2^, a high *V_OC_* of 0.906 V, and FF of 78.9%, leading to a final device PCE of 18.8%, which is one of the highest PCE values for binary OSCs.

**Table 1:** Optimized Photovoltaic Parameters for OSCs.

Active layer^a)^	*J_SC_* [mA cm^−2^]	*J*_cal_ ^b)^ [mA cm^−2^]	*V_OC_* [V]	FF [%]	PCE [%]
PM6:Y6 (DIO)	27.06 (26.88 ± 0.22)	26.36	0.821 (0.820 ± 0.001)	73.9 (73.2 ± 0.6)	16.4 (16.1 ± 0.3)
PM6:Y6 (DIMCH)	26.73 (26.55 ± 0.26)	26.10	0.858 (0.858 ± 0.001)	76.9 (76.6 ± 0.4)	17.6 (17.4 ± 0.1)
PM6:BTP‐ec9 (DIO)	27.53 (27.43 ± 0.18)	26.78	0.828 (0.827 ± 0.002)	76.3 (76.1 ± 0.3)	17.4 (17.3 ± 0.1)
PM6:BTP‐ec9 (DIMCH)	27.13 (26.98 ± 0.23)	26.41	0.862 (0.860 ± 0.001)	77.5 (77.1 ± 0.6)	18.1 (17.9 ± 0.2)
PM6:L8‐BO (DIO)	26.70 (26.58 ± 0.28)	25.68	0.866 (0.864 ± 0.002)	78.4 (78.0 ± 0.5)	18.1 (17.9 ± 0.2)
PM6:L8‐BO (DIMCH)	26.35 (26.22 ± 0.21)	25.37	0.906 (0.905 ± 0.001)	78.9 (78.6 ± 0.3)	18.8 (18.7 ± 0.1)

^a)^ Statistical results from 15 independent devices are listed in parentheses.

^b)^ Current densities derived from EQE plots.

According to formula *E*_loss_ = *E*_g_   − qV_oc_ in Supporting Information, *V*_OC_ of the device is closely related to *E*_loss_ and the decrease of *E*_loss_ is conducive to the increase of *V*_OC_ of the OSCs. Meanwhile, the influence of additives on the *E*_loss_ of different OSCs systems was studied carefully (see Figures S4–S6, Supporting Information, for detailed calculation). Compared with DIO, DIMCH processed devices showed reduced Δ*E*_2_ and Δ*E*_3_ (Figure 1h; Table S1, Supporting Information) in different systems, and the *E*_loss_ of PM6:L8‐BO based OSC modified by DIMCH was calculated to 0.560 eV, slightly lower than that of DIO treated device (0.584 eV). The decrease in *E*_loss_ is estimated to promote *V*_OC_ of device, which was verified in Table 1. As mentioned in the previous article, the residue of additives can cause the energetic disorder of the edge states, leading to an increase in Δ*E*_2_, which can be well avoided by the volatile solid additive DIMCH. Moreover, the decrease in Δ*E*_3_ was due to the rise in EQE_EL_ (Figure 1g), which stemmed from the decrease in crystallinity disparities between donor and acceptor as a result of DIMCH inhibiting excessive aggregation of NFAs, which will be discussed in detail in the next part.

The density functional theory (DFT) simulation was performed to analyze the electrostatic potential distribution and frontier orbital electron distribution of additives of the two additives and photovoltaic materials, and further investigate the aggregation variation for PM6:L8‐BO system. Compared to DIO, DIMCH with a cyclohexyl structure exhibited a more concentrated distribution of positive charges in its core, along with a higher charge distribution density and lower energy level. This indicates that DIMCH had a stronger positive potential than that of DIO. Due to the basic negative and opposite electrostatic potential (ESP) distributions of PM6 and L8‐BO molecules respectively, according to Coulomb's law, DIMCH will form a strong intermolecular attraction with PM6 and repel L8‐BO molecules (Figures S7 and S8, Supporting Information). The presence of DIMCH promoted the formation of larger clusters in PM6, while L8‐BO exhibited a more dispersed distribution, which could balance the crystallinity of the donor and acceptor, and thereby lead to a reduction in *ΔE*_3_.

**Figure 2:**
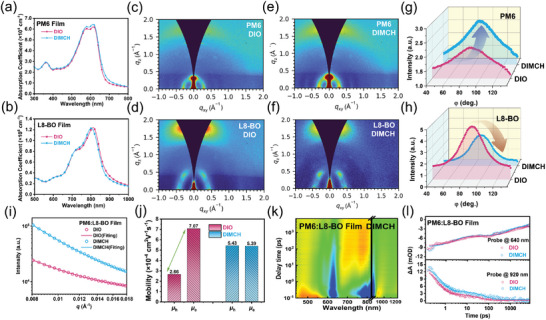
UV–vis spectra of a) PM6 and b) L8‐BO films with DIO or DIMCH. 2D‐GIWAXS patterns of pristine PM6 films with c) DIO or e) DIMCH, and pristine L8‐BO films with d) DIO or f) DIMCH. Pole figures of g) PM6 and h) L8‐BO calculated from the (010) *π*–*π* stacking peak at ≈1.7 Å^−1^. i) The GISAXS intensity profiles and best fittings along the in‐plane direction of PM6:L8‐BO blended films with DIO or DIMCH. j) Hole and electron mobilities of the PM6:L8‐BO films with DIO and DIMCH. k) Color plot of TA spectra of PM6:L8‐BO films under excitation of 800 nm. l) TA dynamics of GSB signals probed at 640 nm and the LE signals probed at 960 nm in the blend film with different additives.

The inhibited NFAs aggregation can also be verified by UV–vis absorption spectroscopy (**Figure** **2**a,b). Compared with that of the DIO‐processed pristine PM6 film, the enhanced 0‐0 shoulder peak indicates the formation of more ordered PM6 packing with the assistance of DIMCH. In contrast, the observed ca. 6 nm blueshifted absorption spectrum for DIMCH processed pristine L8‐BO film suggests the slightly weakened aggregation properties of the NFAs. As for the binary blend (PM6:L8‐BO) film, DIMCH‐processed film showed increased absorption coefficients, but slightly blueshifted absorption peaks compared with DIO treated one (Figure S9, Supporting Information). This further illustrates that DIMCH not only facilitates the PM6 molecular packing but concurrently restricts the aggregation of the L8‐BO to some extent. The changed aggregation behavior of the neat films was further supported by the atomic force microscopy (AFM) images, as depicted in Figure S10 (Supporting Information). In comparison to untreated reference films, PM6 films treated with DIMCH exhibited increased clustering and a coarser morphology, whereas the opposite trend was observed in DIMCH‐treated L8‐BO films, aligning with the findings from UV and ESP analyses.

Grazing‐incidence wide‐angle X‐ray scattering (GIWAXS) visually verified the impact of two additives on molecular stacking and crystallinity of pristine and blend films. Regarding the PM6 films without annealing (Figures S11 and S12, Supporting Information), the (010) *π*–*π* stacking peaks at *q* = 1.68 Å^−1^ with a d‐spacing of 3.74 Å was observed both in out‐of‐plane (OOP) and in‐plane (IP) direction, indicating coexisting of the mixed edge‐on, and face‐on orientation of PM6 neat films. After the incorporation of DIO or DIMCH additive, the more dominated “face‐on” orientation, the smaller d‐spacing values, as well as larger crystalline coherence lengh (CCL) were observed. In detail, the DIMCH‐based annealed films exhibited the largest CCL_010_ of 29.2 Å, accompanied by a tightest *π*–*π* stacking distance of 3.63 Å than that of DIO based annealed films (CCL_010_ = 26.9 Å, *π*–*π* stacking distance = 3.67 Å) and DIMCH based unannealed films (CCL_010_ = 25.4 Å, *π*–*π* stacking distance = 3.68 Å), suggesting the incorporation of DIMCH in PM6 and simutaneously using annealing process can facilitate conspicuously improved molecular ordered stacking during the gradual evaporation of solid additives. We also examined the impact of large content of DIMCH additive on the molecular packing of PM6 films. As shown in Figure S11c,d (Supporting Information) when adding more content of DIMCH (50% wt.), the disordered molecular arrangement appeared in PM6, indicating the adverse effect of excessive additives, and residues.

Similarly, the 2D GIWAXS patterns and line‐cut profiles of L8‐BO films without annealing are shown in Figure S13 (Supporting Information). Both L8‐BO‐based films, with or without DIMCH, showed the same OOP (010) diffraction peaks at ≈1.72 Å^−1^, corresponding to a *π*–*π* stacking distance of 3.65 Å. Following annealing, the (010) *π*–*π* stacking in the OOP direction reduced slightly to 3.63 Å. Furthermore, the (010) *π*–*π* stacking peak in the OOP direction was observed to be broader in the DIMCH‐processed L8‐BO films compared to the control DIO‐processed one, resulting in a wider full width at half maximum (FWHM = 0.20 Å^−1^) and smaller CCL_010_ values (31.4 Å) for the DIMCH‐processed films. In addition, we found that the trends of rDOC (relative degree of crystallinity) variation for the donor and acceptor were different after treatment with the DIO and DIMCH additives. Compared to the rDOC of films treated with DIO, the introduction of DIMCH significantly enhanced the rDOC of the donor, but moderately reduced the rDOC value of the acceptor molecules, which is in accordant with the trend of changed CCL_010_ parameters (Figures S14 and S15 and Table S2, Supporting Information). Considering that the much stronger crystallinity of acceptor than the donor polymer, the crystallinity changes brought about by DIMCH help alleviate the crystallinity difference between the donor and acceptor, which can result in balanced charge transport and suppressed nongeminate recombination, thus leading to the mitigated *ΔE*_3_ in OSCs.

Although more ordered molecular packing can be observed in DIMCH processed blend films, it is difficult to distinguish the stacking properties and domain size of the donor and acceptor in the GIWAXS patterns (Figure S14, Supporting Information). Therefore, the grazing‐incidence small‐angle X‐ray scattering (GISAXS) measurements were used to investigate the phase separation within these binary blend systems by using the Debye–Anderson–Brumberger model (Figure 2i; Figure S16, Supporting Information). The intermixing domain spacings are determined to be 32.9 and 25.6 nm for DIO and DIMCH processed blend, respectively. Furthermore, compared with that of DIO‐processed blend (23.2 nm), the smaller domain size (17.2 nm) of acceptor phase can be obtained in DIMCH‐processed blend, indicating the designed volatile solid additive can inhibit the excessive aggregation of L8‐BO, thereby achieving a more balanced crystallinity of donor and acceptor, and more uniform distribution of morphology structure (Table S3, Supporting Information). According to the atomic force microscopy (AFM) images (Figure S17, Supporting Information), DIMCH processed blends showed a more uniform nanofiber structure, as well as a smaller root‐mean‐square roughness (RMS = 1.16 nm), suggesting a high degree of compatibility and smaller crystallinity disparity of donor and acceptor. Such homogeneous morphology and mitigated crystallinity difference in DIMCH treated PM6:L8‐BO film could be a critical factor to effectively reduce Δ*E*_3_.

Theoretically speaking, the improved crystallinity can promote charge transfer across the active layers. Therefore, the averaged hole (µ_h_) and electron (µ_e_) mobilities were investigated via space‐charge‐limited current (SCLC) method, as depicted in Figure S18 (Supporting Information). The *γ* factor (charge balance factor) in PM6:L8‐BO blend films with DIO and DIMCH were calculated to 2.66 and 0.99, respectively (Figure 2j). According to Equation S3 (Supporting Information), the closer the *γ* value is to 1, the more favorable the increase of EQE_EL_, thus the decrease of Δ*E*_3_. Considering that the unbalanced mobility of carriers can affect the exciton recombination, we investigated the dependence of current density and voltage on light intensity (*P*_light_) for a variety of OSCs systems, which were described as *J_SC_* ∝*P*_light_^𝛼^ and *V_OC_* ∝*nkT*/q ln *P*_light_, respectively. The factor 𝛼 and n of PM6:L8‐BO devices with DIMCH were calculated to be 0.99 and 1.02, respectively (Figure S19, Supporting Information), which were closer to 1 than that of PM6:L8‐BO devices with DIO (0.98 and 1.04), indicating the considerably suppressed bimolecular and geminated recombination, and efficient free charge collection. Moreover, the devices with DIMCH also showed the same trend in the PM6:Y6 and PM6: BTP‐ec9 systems (Figures S20 and S21, Supporting Information).

Transient absorption (TA) spectroscopy was conducted to further probe the charge‐transfer behavior. The pump wavelength of 800 nm was used to selectively excite the acceptor and investigate the process of hole transfer, and the TA spectra of all blends processed with DIO and with DIMCH are shown in Figure 2k,l and Figure S22 (Supporting Information). The signal ranging from 610 to 650 nm was assigned to the ground state bleach (GSB) of PM6, and the signal ranging from 750 to 800 nm was assigned to GSB signal of L8‐BO. Additionally, the excited‐state absorption signals of local excited (LE) state at ≈920 nm was also observed in blend films, which aligned with previous reported work.^[^ ^46^, ^47^, ^48^ ^]^ For the two blend films, the fast decay of L8‐BO GSB signal after the photo excitation and a PM6 bleach signal emerged within 10 ps, which reflected the population and depopulation process of the total excitonic and electronic excitations associated with hole transfer. Afterward, as shown in Figure S23 (Supporting Information), we retrieved the kinetic traces of the two blend films at 640 nm by bi‐exponential function fitting, where *τ*_1_ and *τ*_2_ represented the ultrafast interfacial hole‐transfer process and diffusion of excitons in the acceptor phase toward interface before dissociation, respectively. *τ*_1_ and *τ*_2_ of the DIMCH‐processed PM6:L8‐BO film were 2.54 and 916 ps, respectively, which was much faster than that of DIO‐processed film (3.32 and 1130 ps). This result manifested that the addition of DIMCH additive remarkably facilitated hole transfer, which benefitted charge generation in the corresponding OSCs. On the other hand, the decay lifetime of LE signal at 920 nm in the DIMCH‐processed film (*τ*_1_ of 0.65 ps and *τ*_2_ of 73 ps) was obviously slower than that of the blend film with DIO (*τ*_1_ of 0.52 ps and *τ*_2_ of 36 ps), indicating much slower non‐geminate recombination process in the former. In DIMCH‐treated devices, the enhanced hole transfer and reduced recombination can yield higher EQE_EL_, thereby leading to a substantial decrease in Δ*E*_3_.^[^ ^32^, ^33^, ^34^ ^]^

**Figure 3:**
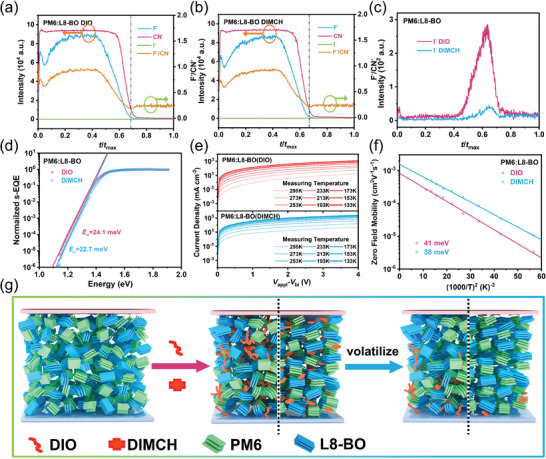
Relative TOF‐SIMS ion intensity of F^−^, CN^−^, F^−^/CN^−^, and I^−^ based on a) DIO treated and b) DIMCH treated PM6:L8‐BO BHJ films. c) Intensity ratio as a function of *t*/*t*_max_ in PM6:L8‐BO BHJ films with DIO or DIMCH additive. *t* and *t*_max_ are the specific and total sputtering time, respectively. d) s‐EQE of PM6:L8‐BO‐based devices with DIO and DIMCH at absorption onset. e) Temperature‐dependent SCLC curves for PM6:L8‐BO‐based hole‐only devices. f) Hole mobility of corresponding devices as a function of 1/T^2^ using SCLC estimated data. g) Schematic diagram of vertical distribution of donor and acceptor in BHJ films with DIO or DIMCH additive.

Although the mechanism of DIMCH addition on Δ*E*_3_ is thoroughly studied, the difference in the effects of the two additives on Δ*E*_2_ remains unclear. To this end, time‐of‐flight secondary ion mass spectrometry (TOF‐SIMS) was further performed on PM6:L8‐BO blend films. In view of chemical structures of molecules, the signal of I^−^ can track the change of DIO and DIMCH, L8‐BO can be represented by the signal of CN^−^, whereas F^−^ that existing in both PM6 and L8‐BO can represent the total amount of light‐harvesting components. As displayed in **Figure** **3**a,b, the amount of both additives was very small relative to the donor or acceptor, indicating that most of DIMCH additives were removed during the spin coating or the thermal annealing process. However, in enlarged curve (Figure 3c), there was still a large amount of DIO remaining, mainly distributed in the bottom of the film. In contrast, DIMCH almost disappeared from blend films after post‐treatment, indicating volatile additive worked during film formation process, and avoided adverse effect of residues. We also conducted transmission electron microscope‐energy dispersive spectroscopy (TEM‐EDS) to investigate the residue of additives in PM6:L8‐BO after annealing. The results, depicted in Figure S24 (Supporting Information), indicated that DIO‐treated PM6:L8‐BO retained 7.8% iodine content, whereas DIMCH‐treated films exhibited no iodine signals, corroborating the complete disappearance of DIMCH after annealing process. In addition, the distribution of donor and acceptor in the vertical direction can also be roughly estimated by comparing the intensity ratio of F^−^/CN^−^. As shown in the Figure 3a,b, the F^−^/CN^−^ value of the mixed film with DIMCH near the bottom (that is, the active layer in the device near the anode part of the cell) was 0.20, slightly larger than that of the mixed film with DIO (0.18), which indicated PM6 was enriched near the anode after DIMCH addition, theoretically facilitating charge transport, and inhibiting charge recombination.

As mentioned earlier, the residue of additives can cause energetic disorder in the edge states, resulting in an increase in Δ*E*_2_. Here, highly sensitive external quantum efficiency (s‐EQE) measurements were performed on PM6:L8‐BO‐based devices with additive modification to investigate their impact on energy disorder.^[^ ^49^, ^50^ ^]^ By linear fitting of the s‐EQE spectra beyond the bandgap edge (Figure 3d), the Urbach energy (*E*_u_) values were calculated to be 24.1 and 22.7 meV for PM6:L8‐BO‐based devices with DIO and DIMCH, respectively. The smaller *E*_u_ implied reduced energetic disorder in the PM6:L8‐BO‐based devices with DIMCH, which was attributed to the ordered packing of the materials in blend films and reduction of additive residues. Moreover, the addition of DIMCH resulted in the decrease of the device *E*_u_ (Figures S5 and S6, Supporting Information) in multiple OSCs systems. The energetic disorder (𝜎) was also estimated by temperature‐dependent hole transport measurement (Figure 3e,f; Table S4, Supporting Information) using Gauss disorder model (GDM). Figure 3c depicted the plots of hole zero‐field mobilities of two devices as a function of 1/*T* ^2^ and the corresponding fitted straight curves.^[^ ^51^ ^]^ The decreasing 𝜎 values from 41 meV (DIO) to 38 meV (DIMCH) indicated that less trapped states in the PM6:L8‐BO‐based devices with DIMCH. The above tests further verified that the addition of DIMCH led to the decrease of device energy disorder and contributed to the reduction of Δ*E*_2_.

With extensive characterizations of individual PM6 and L8‐BO systems, as well as the PM6:L8‐BO BHJ blend, we present a schematic mechanism for the formation of the state‐of‐art morphology utilizing the DIMCH additive. As depicted in Figure 3g, when the PM6:L8‐BO series films were processed with DIO, the blend shows distinct large‐scale phase separation due to the rapid evaporation of solvent and mismatched crystallinity properties of donor and acceptor. Furthermore, the prolonged presence of DIO in the film, owing to its high boiling point, leads to an increased trap density in blend film, and consequently results in higher energy losses in the DIO‐processed devices. By replacing DIO with volatile solid additive DIMCH, the DIMCH with concentrated cyclohexane group weaken the disparity of imbalanced crystallinity of the PM6 and L8‐BO, lead to a uniform, and homogeneous network structure of crystal domain with reduced D–A phase separation. The highly volatile nature of DIMCH prevents the residue of additives in PM6:L8‐BO film, promoting the ordered molecular packing, and effectively suppressing energy disorder of the edge state and trap states, therefore reducing radiative/nonradiative recombination induced *E_l_*_oss_.

## 3 Conclusion

In conclusion, a new volatile solid additive DIMCH was explored and it functions as a morphology‐directing guest to effectively reduce energy loss in multiple state‐of‐art photovoltaic systems (PM6:Y6, PM6: BTP‐ec9, and PM6:L8‐BO), leading to one of the highest efficiencies of 18.8% in binary OSCs. DIMCH with the unique cyclohexyl group possesses a more concentrated electrostatic potential distribution compared to DIO featured with a straight alkyl group, which effectively facilitates the interaction with PM6 and improves its crystallinity. Moreover, DIMCH effectively inhibited the excessive aggregation of L8‐BO to weaken the disparity of imbalanced crystallinity of the PM6 and L8‐BO, leading to a uniform and homogeneous network structure of crystal domain with reduced D–A phase separation, thus, promoting charge transport, and inhibiting radiative/non‐radiative recombination. Meanwhile, the volatilization characteristics of DIMCH suppressed energy disorder of the edge state and trap states, which is beneficial for obtaining simultaneously mitigated Δ*E*_2_ and Δ*E*_3_ in solar cell devices. Although there are still lots of questions remaining for the basic mechanism of reducing *E*_loss_, our study elaborates the relationship between morphology, trap density, and *E*_loss_ with the assisted by the volatile DIMCH solid additive, we believe these findings are extremely important in the understanding of how to design solid additive to optimize the blend morphology and reduce *E*_loss_ of current state‐of‐art OSCs toward higher performance.

## Conflict of Interest

The authors declare no conflict of interest

## Supporting information

Supporting Information

Supporting Information

## Data Availability

The data that support the findings of this study are available from the corresponding author upon reasonable request.
