# Supplementary material for: Tackling Energy Loss in Organic Solar Cells via Volatile Solid Additive Strategy
Source: Adv Sci (Weinh). 2024 Apr 18;11(25):2401330. doi: 10.1002/advs.202401330 (PMC11220641; doi:10.1002/advs.202401330)
Supplement: Supplementary file 1 — Supporting Information [file ADVS-11-2401330-s001.pdf]

ADVANCED SCIENCE
Open Access

Supporting Information

for *Adv. Sci.*, DOI 10.1002/advs.202401330

Tackling Energy Loss in Organic Solar Cells via Volatile Solid Additive Strategy

Huimin Xiang, Fengbo Sun, Xufan Zheng, Bowen Gao, Panpan Zhu, Tingting Cong, Yuda Li, Xunchang Wang and Renqiang Yang**

Supporting Information

Tackling energy loss in organic solar cells via volatile solid additive strategy

Huimin Xiang, Fengbo Sun, Xufan Zheng, Bowen Gao, Panpan Zhu, Tingting Cong, Yuda Li, Xunchang Wang, Renqiang Yang**

H. Xiang, F. Sun, X. Zheng, P. Zhu, T. Cong, X. Wang, Prof. Renqiang Yang
School of Optoelectronic Materials and Technology, Jianghan University, Wuhan 430056, China
E-mail: yangrq@jhun.edu.cn, wangxc@jhun.edu.cn

B. Gao, Y. Li
Key Laboratory for Green Process of Ministry of Education, School of Chemical Engineering and Pharmacy, Wuhan Institute of Technology, Wuhan 430205, China

Materials and Methods

Materials

Chloroform (Sigma-Aldrich, ≥99%), 1,8-diiodioctane (DIO, Sigma-Aldrich, 98%) and 1,4-Cyclohexanedimethanol (CAS:105-08-8, Bidepharm, 99%) were purchased from commercial sources and used without further purification. PM6, Y6, BTP-ec9, L8-BO and PDINN were purchased from Organtec Ltd. The detailed synthetic procedures are as follows.

Synthesis of DIMCH

In brief, triphenylphosphine (6 eq.) and imidazole (6 eq.) were dissolved in 30 ml of dichloromethane and stirred until homogeneity. Iodine (6 eq.) was added slowly in portion in above solution and the resulted mixture was stirred until all iodine granules were dissolved. 1,4-Cyclohexanedimethanol was then slowly added in as prepared solution and stirred overnight at room temperature under nitrogen atmosphere to obtain brown solution. Afterwards, the brown solution was diluted with hexane and filtered to remove the solid residues, and then concentrated under reduced pressure to obtain yellow solid. The pale yellow solid was washed with hexane and filtered twice. The filtrate liquid was removed under reduced pressure and the crude product was purified by flash chromatography with silica gel and hexane eluent to purify DIMCH product. In order to obtain pure DIMCH, the product was further heated in vacuo to remove the low-boiling impurities. As a result, a white solid of DIMCH was obtained (Yield 47%). ^{1}H NMR (400 MHz, $CDCl_3$) δ 3.10 (d, 4H), 1.92 (d, 4H),

1.39 (s, 2H), 1.04 (m, 4H). ^{13}C NMR (101 MHz, $CDCl_3$) δ 77.34, 77.03, 76.71, 39.64, 32.79, 14.98.

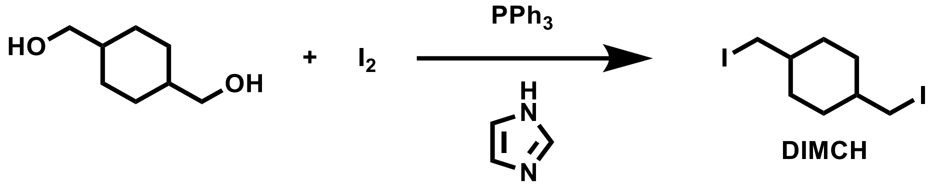

Scheme S1. The synthetic routes of DIMCH.

Methods

Nuclear magnetic resonance (NMR) spectroscopy: ^1H and ^{13}C NMR spectra were measured on a Bruker AV-500 MHz spectrometer in deuterated solvents at room temperature. Chemical shifts were recorded with tetramethylsilane (TMS) as the internal reference.

UV-vis absorption spectra: The UV–vis absorption spectra of solutions and films were recorded using a Hitachi U-4100 spectrophotometer.

GIWAXS characterization: 2D-GIWAXS experiments were carried out on a GANESHA 300XL+ system from JJ X-ray. The instrument is equipped with a Pilatus 300K detector, with pixel size of 172 × 172 μm. The X-ray source is a Genix 3D M icrofocus sealed Tube X-Ray Cu-source with integrated Monochromator (30 W). The wavelength used is λ = 1.5418 Å. The detector moves in a vacuum chamber with sample-to-detector distance varied between 0.115 m and 1.47 m depending on the configuration used, as calibrated using silver behenate (d001 = 58.380 Å). The minimized background scattering plus high-performance detector allows for a detectable q-range varying from 3×10^{-3} to 3 $Å^{-1}$ (0.2 to 210 nm). The sample was placed vertically on the goniometer and tilted to a glancing angle of 0.2° with respect to the incoming beam. A small beam was used to get a better resolution. The accumulation time was 30 minutes for each measurement. In-plane and out-of-plane line-cuts were obtained using SAXSGUI program.

Atomic force microscopy (AFM): Standard tapping-mode AFM measurements in ambient were performed on a Scanned Probe Imaging and Development (SPID) on Park NX-10. The AFM images were confirmed from different samples and scan areas. The root-mean-square roughness (RMS) values of the height images were obtained from the whole scan area (2 μm × 2 μm). All the AFM images were flattened and exported from the software.

Surface Electrostatic Potential (ESP) and Molecular Polarity Index: We performed quantum chemical calculations with the Gaussian 09 software package by selecting the 6-31G (d, p) basis set. The ground-state geometries were optimized by virtue of the popular B3LYP exchange-correlation functional. The ESP analysis was carried out by a wavefunction analysis tool Multiwfn. The surface electrostatic potential

distribution diagrams of the non-fullerene acceptors were simulated with an electron density of 0.001 a.u. Molecular polarity index (MPI) of the acceptor molecules was calculated on the basis of the distribution characteristics of the ESP on the molecular surface. The value of MPI can be given through the following formula:

$$\mathrm{MPI} = (1/A)\iint |V(r)| dS$$

where V is the electrostatic potential of the molecule, integration is the integration of the molecular surface S, and A is the molecular surface area. The greater the MPI, the greater the overall polarity of the molecule. Because the non-uniformity of the charge distribution in the system is a manifestation of the polarity of the molecules, the more uneven the distribution, the more positive or negative regions of the electrostatic potential on the surface of the molecule will appear, making the MPI larger. The molecular polar surface area is the area where the absolute value of the ESP is greater than 10 kcal/mol, and the molecular non-polar surface area is the area where the absolute value of the ESP is less than 10 kcal/mol.

Femtosecond Transient Absorption Spectroscopy Measurements: Femtosecond transient absorption spectroscopy measurements were performed on an Ultrafast Helios pump-probe system in collaboration with a regenerative amplified laser system from Coherent. An 800 nm pulse with a repetition rate of 1k Hz, a length of 100 fs, and an energy of 7 μJ cm^{-2} $pulse^{-1}$, was generated by an Ti:sapphire amplifier (Astrella, Coherent). Then the 800 nm pulse was separated into two parts by a beam splitter. One part was coupled into an optical parametric amplifier (TOPAS, Coherent) to generate the pump pulses at various wavelength. The other part was focused onto a sapphire plate and a YAG plate to generate white light supercontinuum as the probe beams with spectra covering 440-820 nm and 820-1200 nm, respectively. The time delay between pump and probe was controlled by a motorized optical delay line with a maximum delay time of 8 ns. The pump pulse is chopped by a mechanical chopper with 500 Hz and then focused on to the mounted sample with probe beams. The probe beam was collimated and focused into a fiber-coupled multichannel spectrometer with CCD sensor. The energy of pump pulse was measured and calibrated by a power meter (PM400, Thorlabs). The samples used for TA measurements were obtained by spin-coating the blend solutions on the quartz substrates.

Transmission electron microscopy energy dispersive x-ray spectroscopy (TEM-EDS): In order to study the variation in chemical composition in bulk films, TEM-EDS (FEI Tecnai F20) analysis was performed at an accelerating voltage of 200 kV using a FEI G^2 Tecnai.

Device characterization: The current-voltage (*J-V*) characteristics were measured with a Keithley 2450 source measurement unit. The OSCs were measured under an irradiation intensity of 100 mW/cm^2 (AM 1.5 G) by a Newport solar simulator. The effective area of the device is 0.042 cm^2. The EQE spectra were analyzed using an integrated system (LST-QE). The high sensitive EQE was measured by using an integrated system,

where the photocurrent was amplified and modulated by a lock-in instrument.

Fabrication and characterization of single-carrier devices: The hole and electron mobilities were calculated using the space charge limited current (SCLC) model with a device configuration of ITO/PEDOT: PSS/active layer/MoO_3/Ag and ITO/ZnO/active layer/PDINN/Ag, respectively, where the current density is calculated by:

$$u = \frac{8}{9} \cdot \left(\frac{\sqrt{J}}{V} \right)^2 \cdot \frac{d^3}{\varepsilon_0 \varepsilon_r}$$

where J stands for current density, ε_o is the permittivity of free space, ε_r is the relative dielectric constant of the transport medium, μ is the hole mobility, V is the voltage drop across the device ($V = V_{appl} - V_{bi} - V_{RS}$, where V_{appl} is the applied voltage to the device, V_{bi} is the built-in voltage due to the difference in work function of the two electrodes, and V_{RS} is the voltage drop due to series resistance across the electrodes), and L is the thickness of the active layer.

Energy loss Calculation: The total energy loss (E_{loss}) can be attributed to three components following the equations below:

$$\begin{aligned} E_{loss} &= E_g - qV_{oc} \qquad (1) \\ &= \left(E_g - qV_{oc}^{SQ}\right) + \left(qV_{oc}^{SQ} - qV_{oc}^{rad}\right) + \left(qV_{oc}^{rad} - qV_{oc}\right) \\ &= \left(E_g - qV_{oc}^{SQ}\right) + q\Delta V_{oc}^{rad,below\ gap} + q\Delta V_{oc}^{non-rad} \\ &= \Delta E_1 + \Delta E_2 + \Delta E_3 \end{aligned}$$

where E_g is the bandgap, q is the elementary charge, V_{oc}^{SQ} is the maximum voltage based on the Shockley-Queisser limit (SQ limit), V_{oc}^{rad} is the open-circuit voltage when there is only radiative recombination, $\Delta V_{oc}^{rad,below\ gap}$ is the voltage loss of radiative recombination from the absorption below the bandgap and $\Delta V_{oc}^{non-rad}$ is the voltage loss of non-radiative recombination. ΔE_1 is due to radiative recombination from absorption above the bandgap, ΔE_2 is due to radiative recombination from absorption below the bandgap, and ΔE_3 is due to non-radiative recombination. The third component, the non-radiative loss, could be directly calculated by the following relation:

$$\Delta E_3 = q\Delta V_{oc}^{non-rad} = -kT\ln(EQE_{EL}) \qquad (2)$$

where k is the Boltzmann constant, T is temperature and EQE_{EL} is the radiative quantum efficiency of the OSCs when charge carriers are injected into the device in the dark.

$$EQE_{EL} = \gamma\chi\Phi_{PL}\eta_{out} \qquad (3)$$

Where EQE_{EL} is the electroluminescence quantum efficiency of solar cells via injecting charge carriers into the device under dark, γ is the charge balance factor (typically stipulated to be 1), χ is the fraction of

recombination events due to the radiative decay (the spin-singlet configuration in excitons), Φ_{PL} is the photoluminescence quantum efficiency (equivalent to the luminescence yield of spin-singlet excitons) and η_{out} is the photon out-coupling efficiency (generally evaluated to be 0.3).

Supplementary Figures

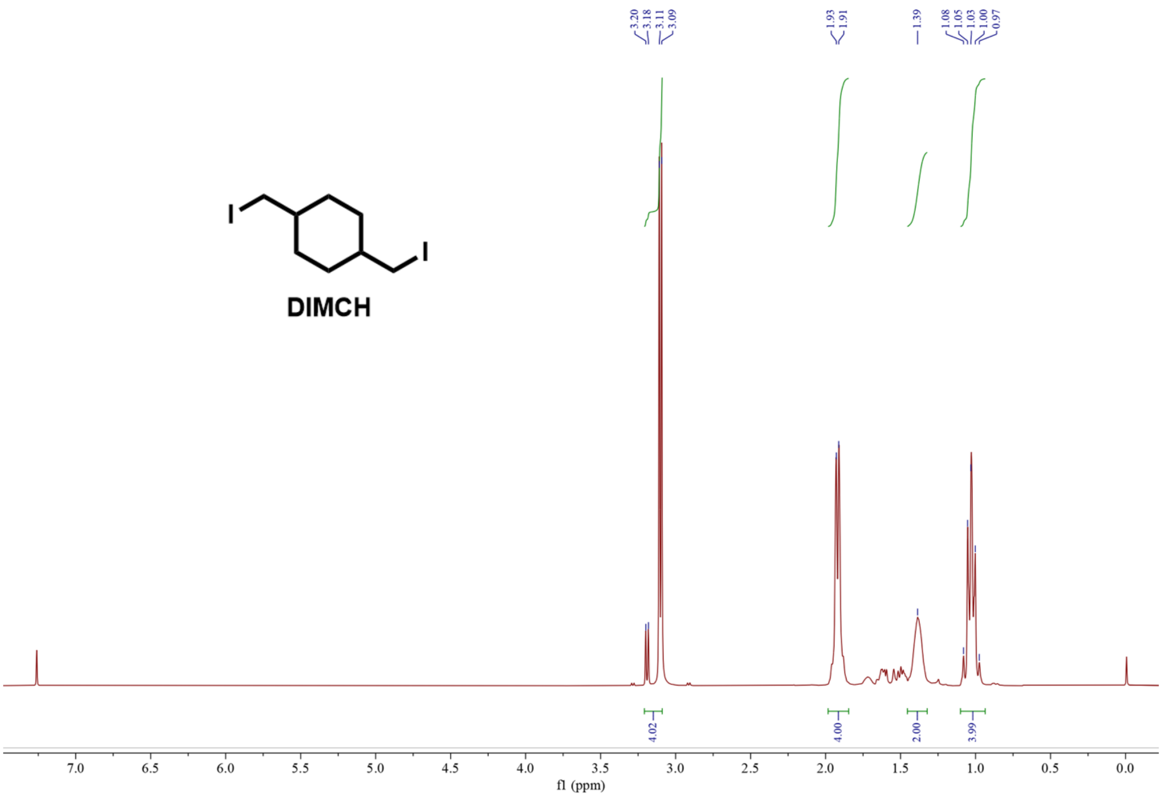

Figure S1. ^{1}HNMR spectra of DIMCH.

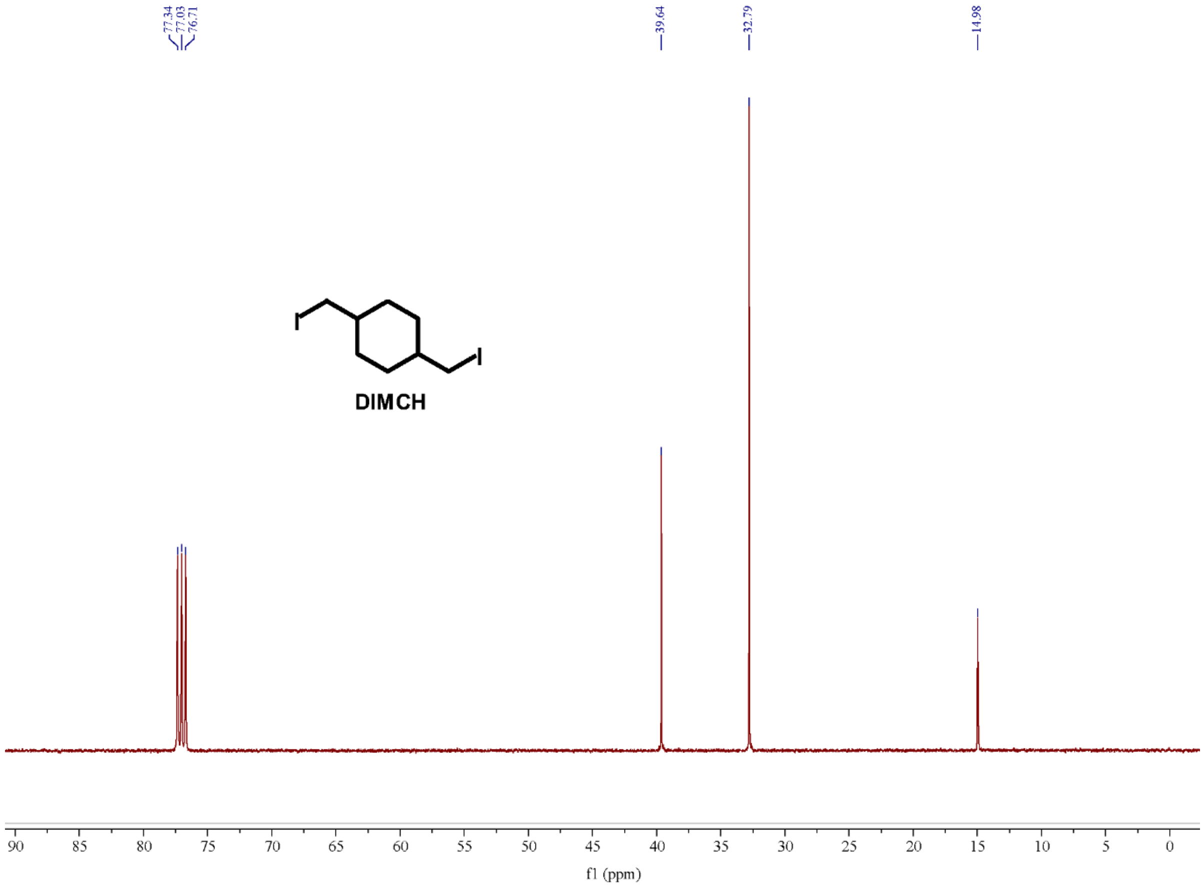

Figure S2. ^{13}CNMR spectra of DIMCH.

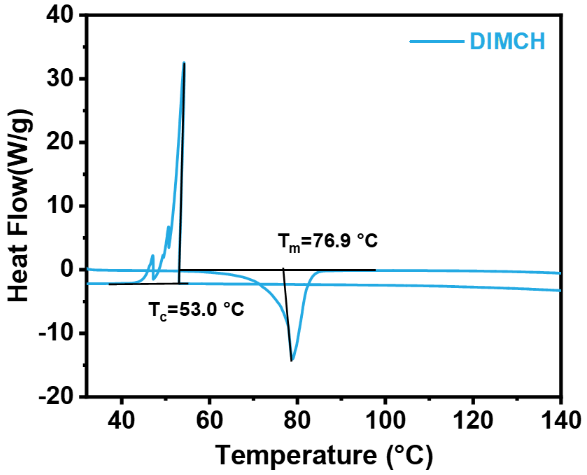

Figure S3. The differential scanning calorimetry (DSC) analyses for the solid additive DIMCH.

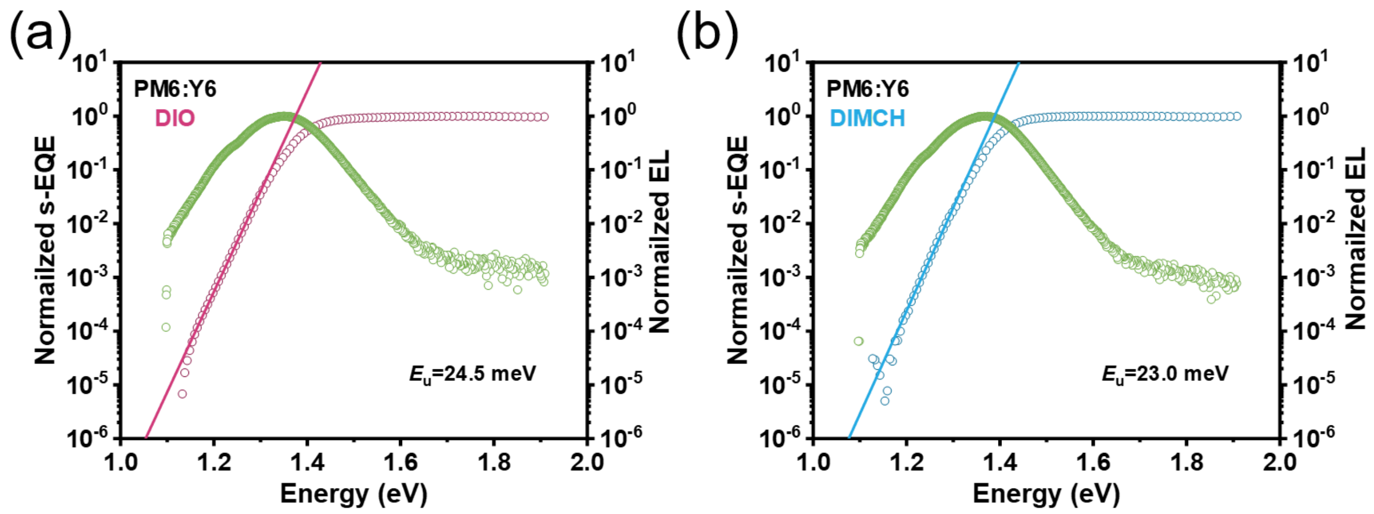

Figure S4. The s-EQE and EL curves of the PM6:Y6-based OSCs with additives modification:(a) DIO, (b) DIMCH.

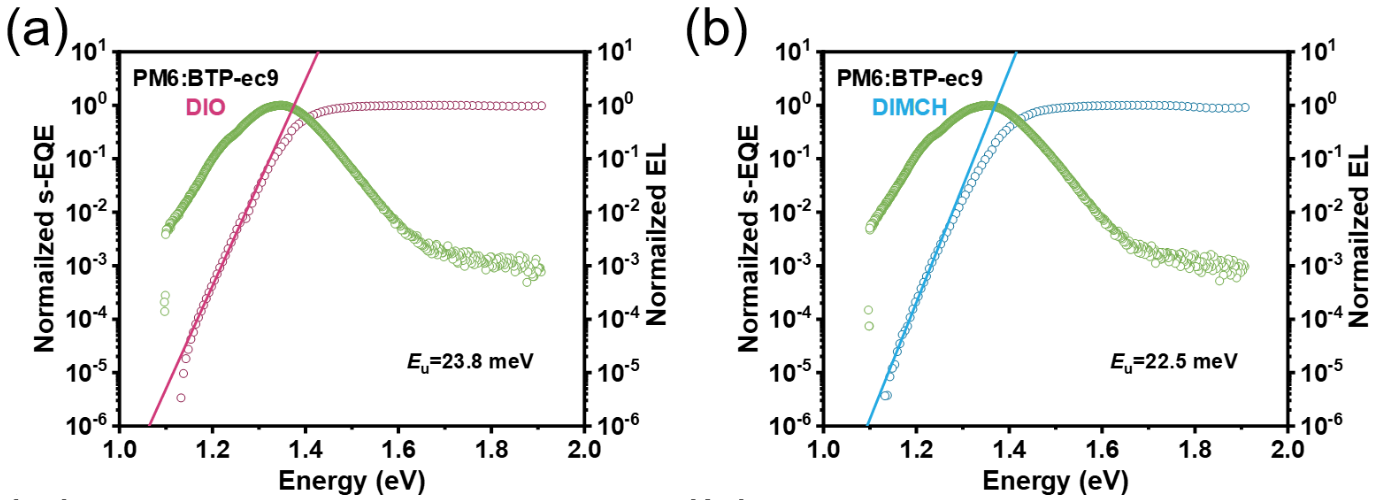

Figure S5. The s-EQE and EL curves of the PM6:BTP-ec9-based OSCs with additives modification:(a) DIO, (b) DIMCH.

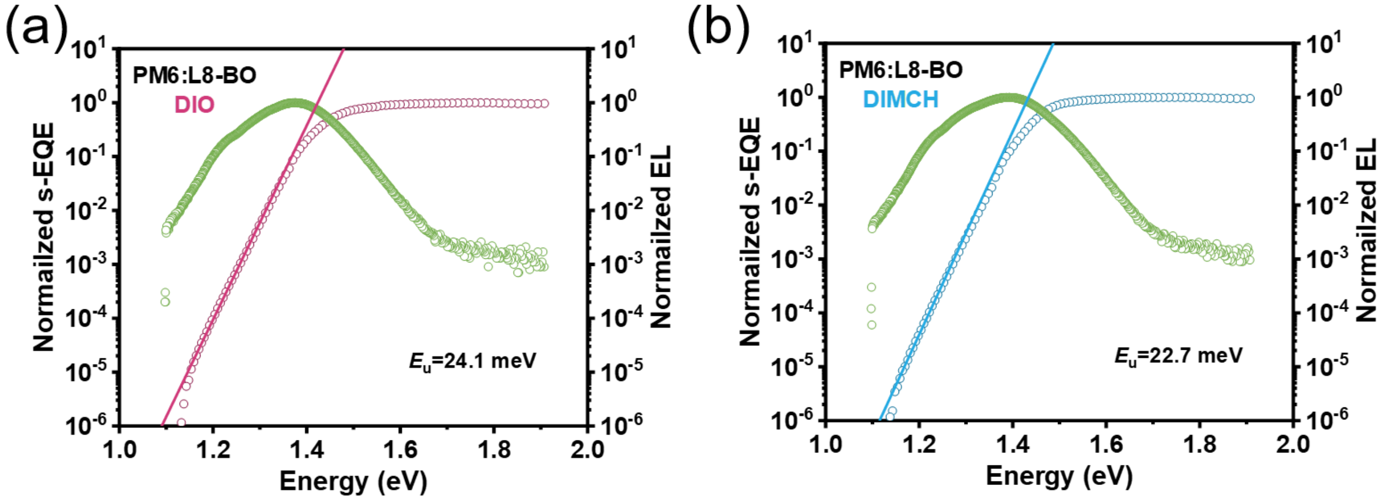

Figure S6. The s-EQE and EL curves of the PM6:L8-BO-based OSCs with additives modification:(a) DIO, (b) DIMCH.

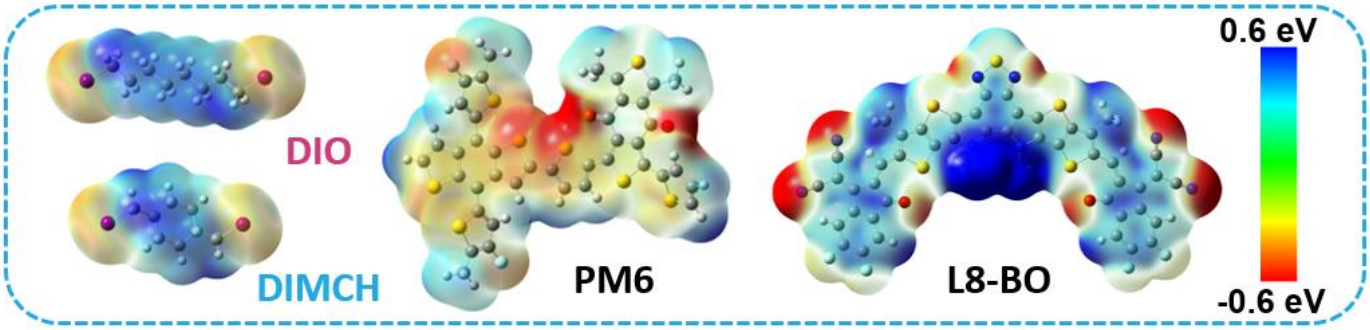

Figure S7. The electrostatic potential (ESP) of the additives DIO, DIMCH and photovoltaic materials PM6 and L8-BO.

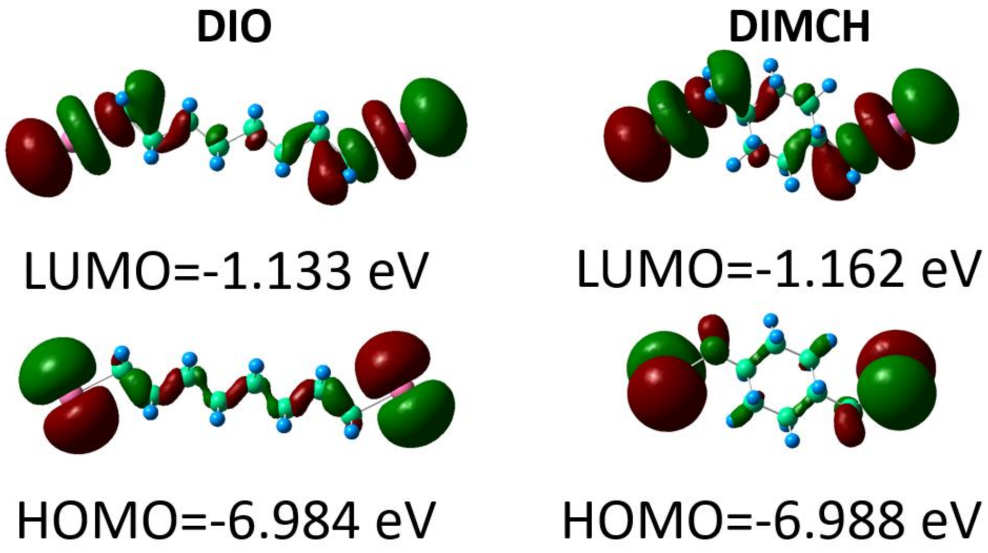

Figure S8. Frontier orbital electron distribution of additives based on DFT simulations.

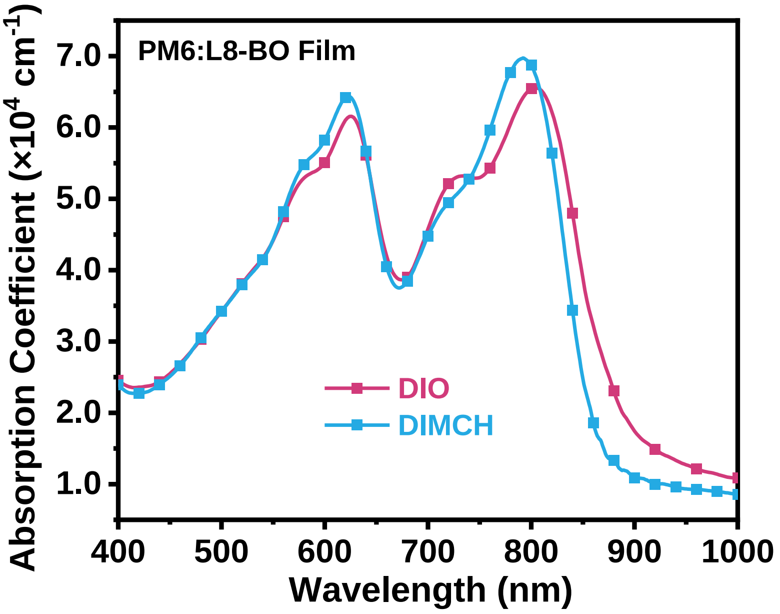

Figure S9. UV−vis spectra of PM6:L8-BO films with DIO or DIMCH.

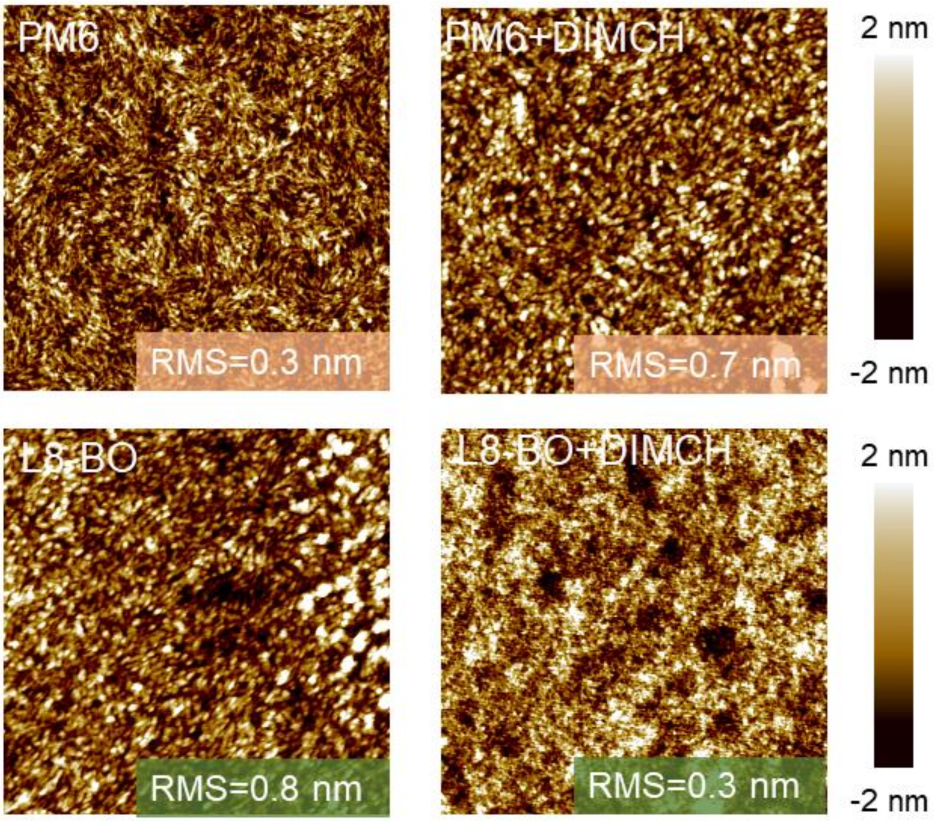

Figure S10. The AFM height images of PM6 neat films (a) with or (b) without DIMCH. The AFM height images of L8-BO neat films (c) with or (d) without DIMCH.

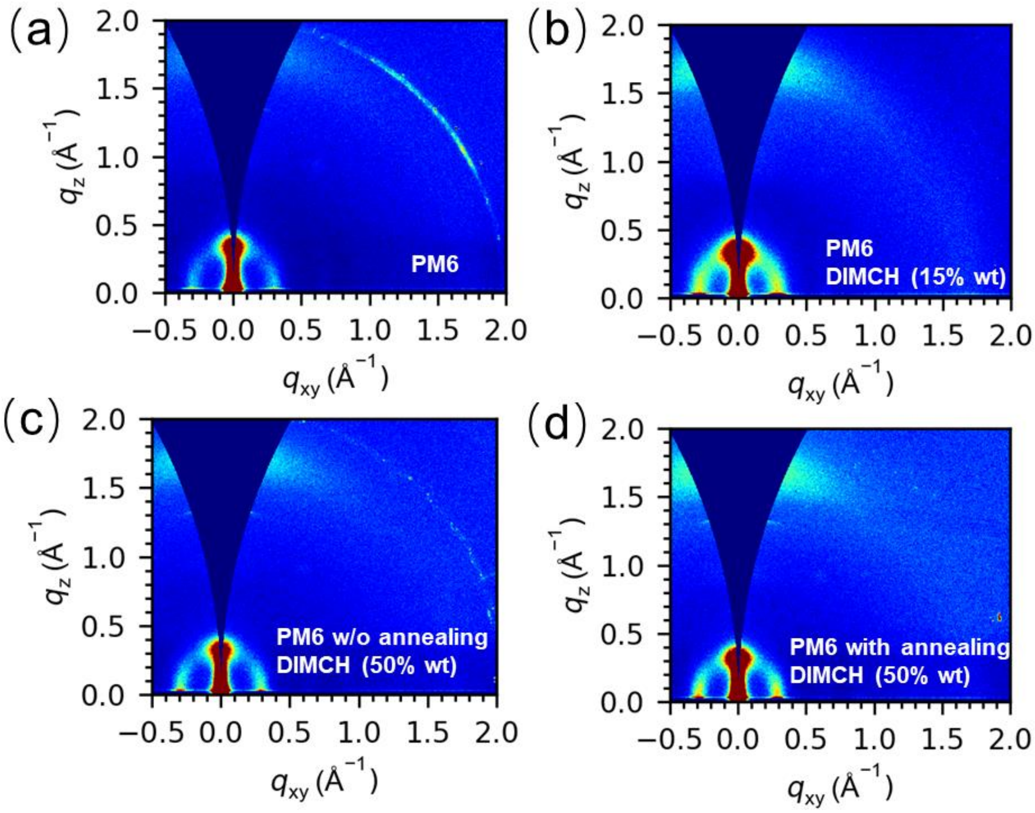

Figure S11. 2D-GIWAXS patterns of the PM6 films before annealing: (a) with and (b) without DIMCH (15% wt). 2D-GIWAXS patterns of the PM6 films with excessive DIMCH (50% wt): (c) without annealing and (d) with annealing.

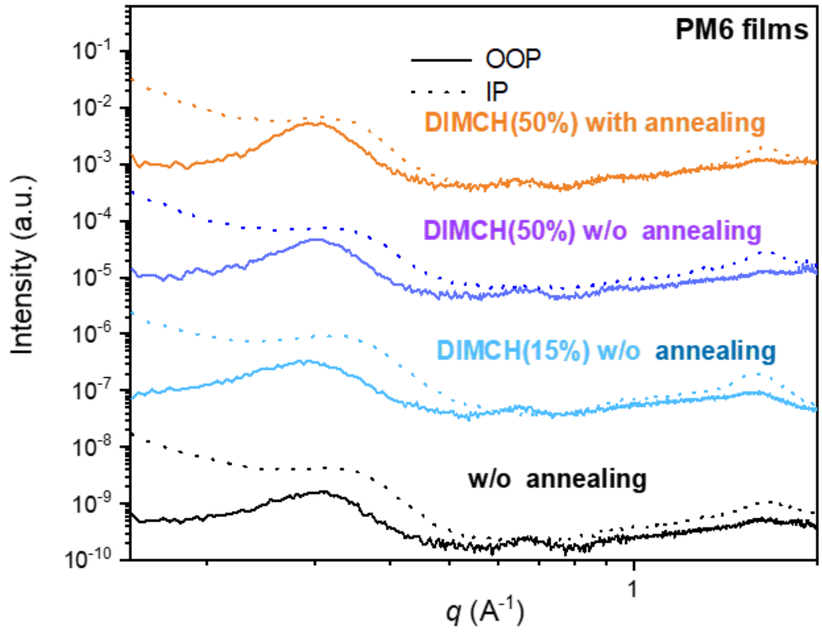

Figure S12. Corresponding line-cut profiles of PM6 films in IP and OOP directions.

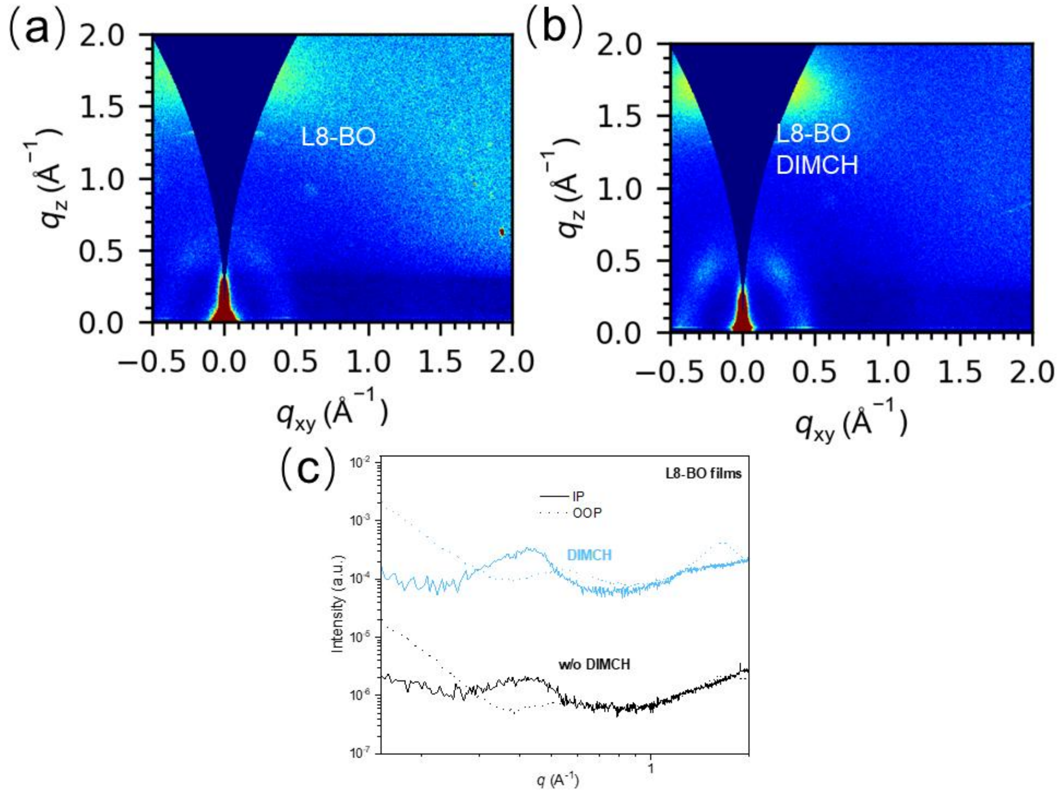

Figure S13. 2D-GIWAXS patterns of the L8-BO films before annealing: (a) with and (b) without DIMCH. (c) Corresponding line-cut profiles of L8-BO films in IP and OOP directions.

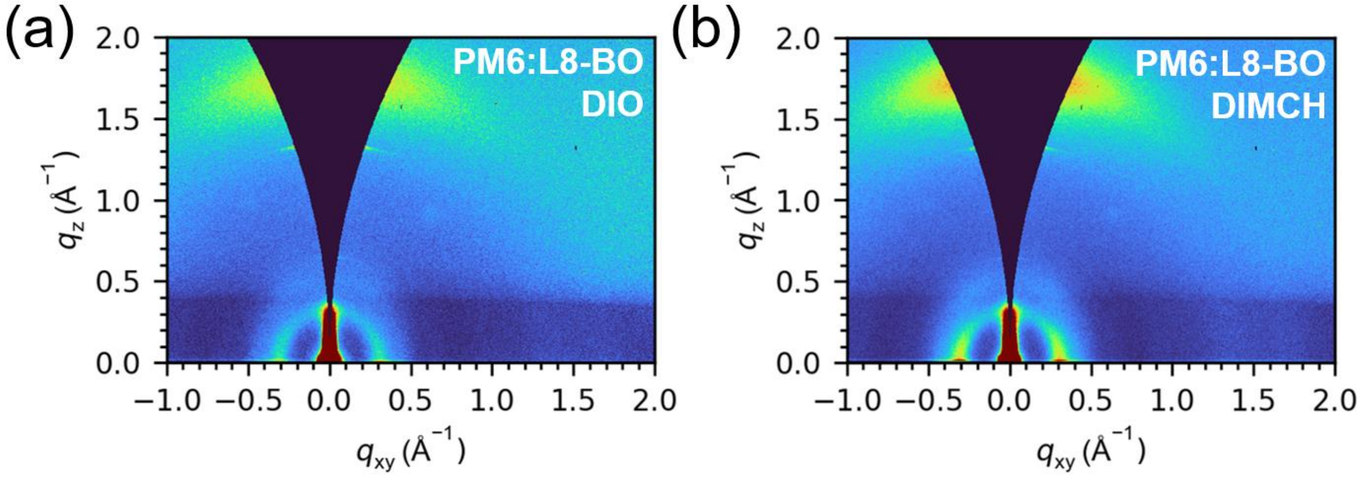

Figure S14. 2D-GIWAXS patterns of the PM6:L8-BO films with (a) DIO or (b) DIMCH. Corresponding line-cut profiles in IP and OOP directions.

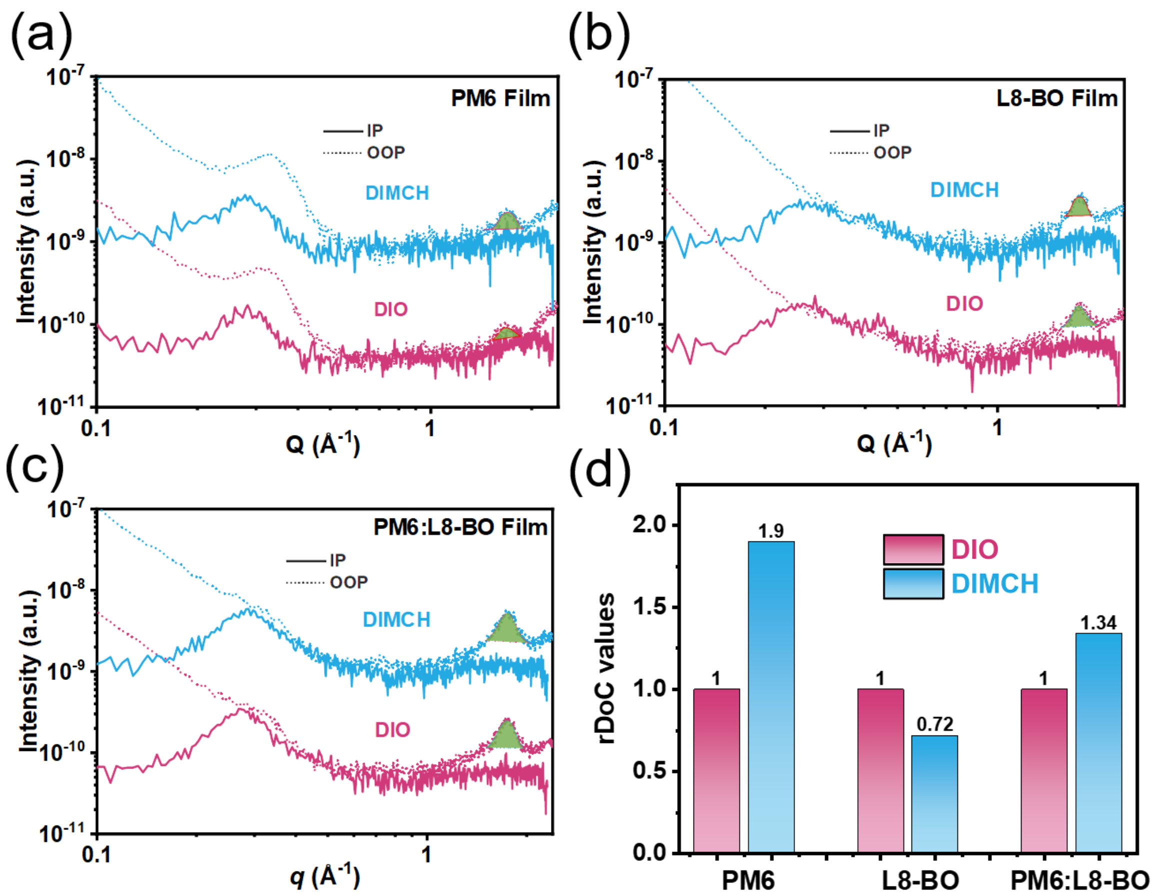

Figure S15. Out-of-plane (dashed line) and in-plane (solid line) line-cut profiles of the GIWAXS patterns for the (a) PM6, (b) L8-BO and (c) PM6:L8-BO films processed with the DIO and DIMCH additives. (d) The rDoC values for the blend films processed with the DIO and DIMCH additives.

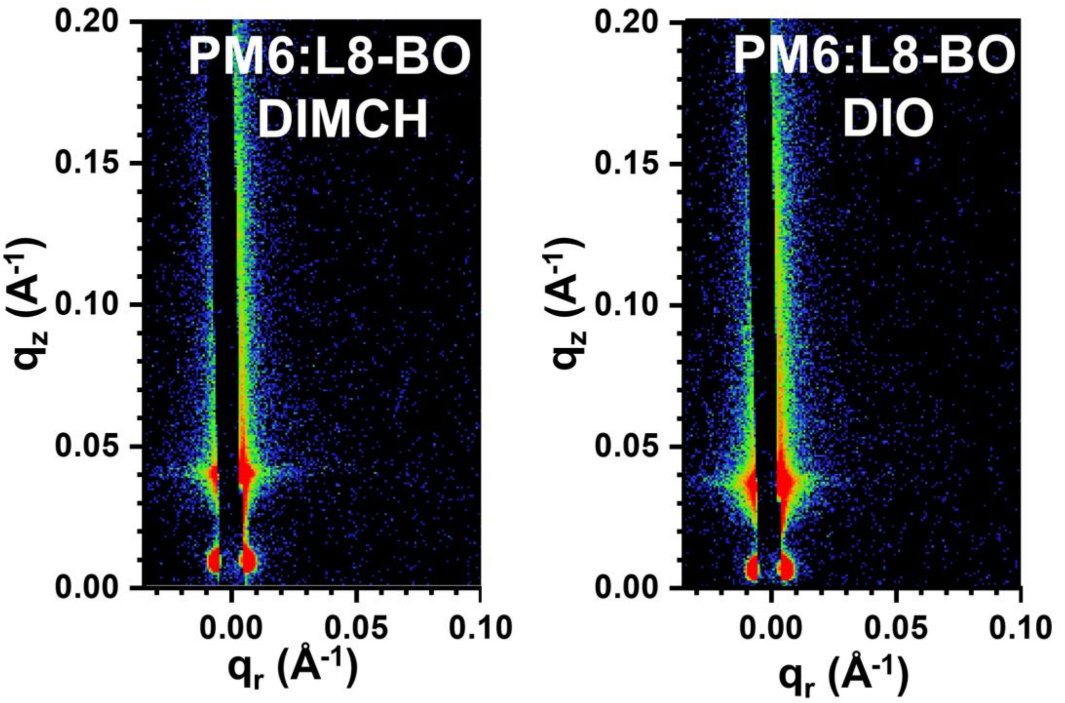

Figure S16. 2D- GISAXS patterns of PM6:L8-BO blended films with DIO or DIMCH.

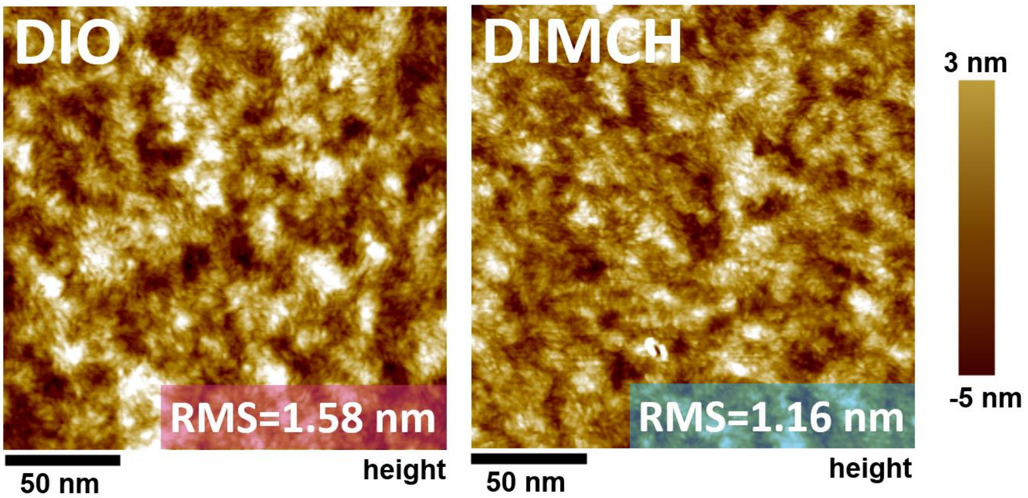

Figure S17. The AFM height and phase images of PM6:L8-BO blended films with DIO or DIMCH.

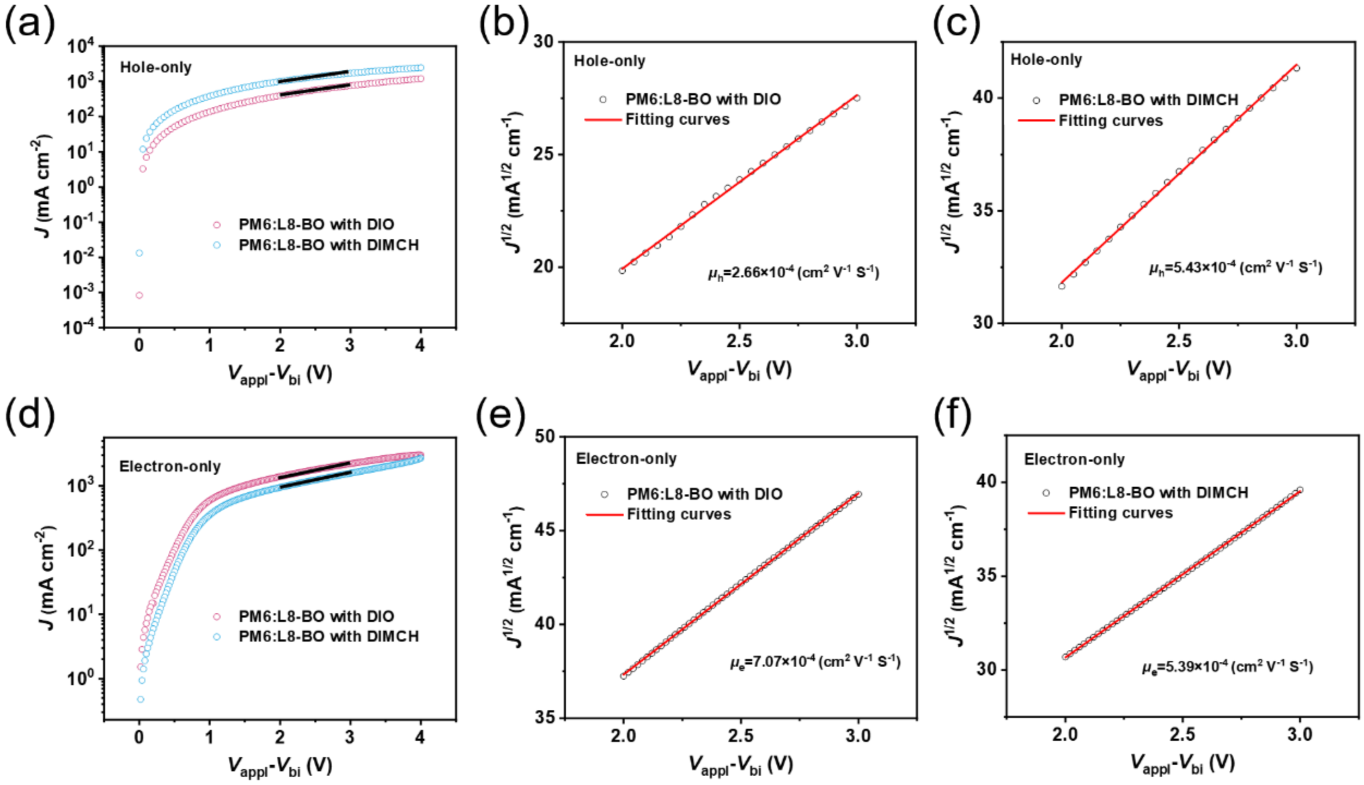

Figure S18. Dark *J-V* characteristics of DIO- and DIMCH-treated optimized PM6:L8:BO blend in (a, b, c) hole-only devices (ITO/PEDOT:PSS/blend film/MoO_3/Ag) and corresponding fitting curves, (e, f, g) electron-only devices (ITO/ZnO/blend film/Ag) and corresponding fitting curves.

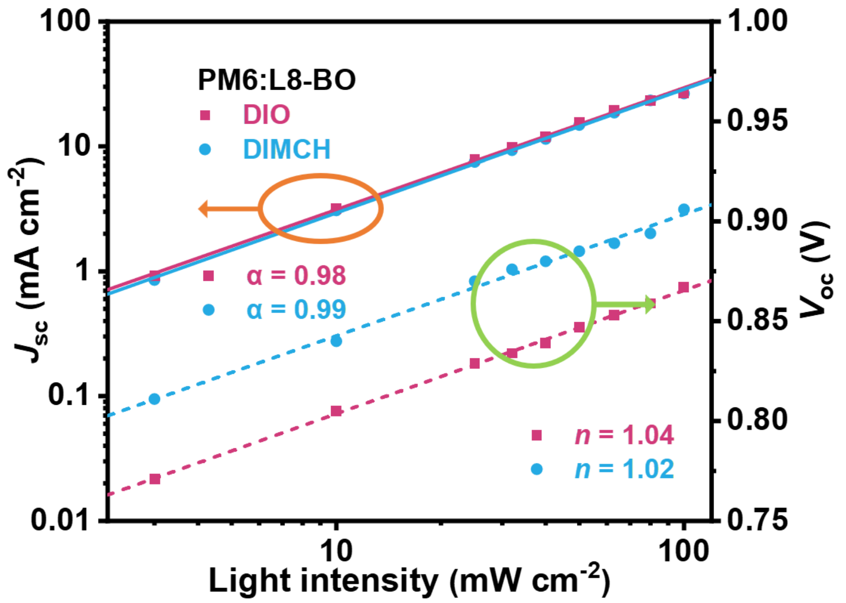

Figure S19. J_{ph} and V_{OC} versus light intensity plots of the PM6:L8-BO-based OSCs.

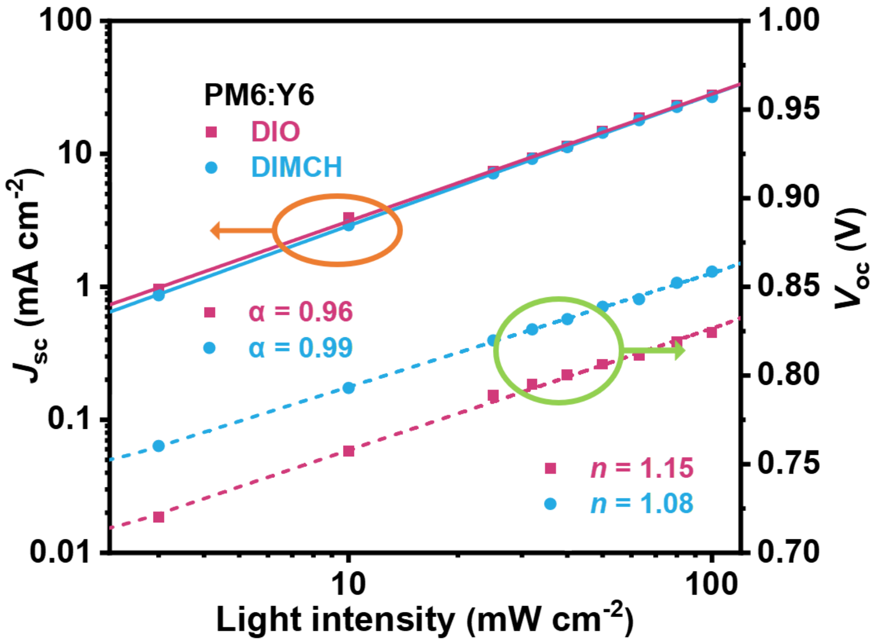

Figure S20. J_{ph} and V_{OC} versus light intensity plots of the PM6:Y6-based OSCs.

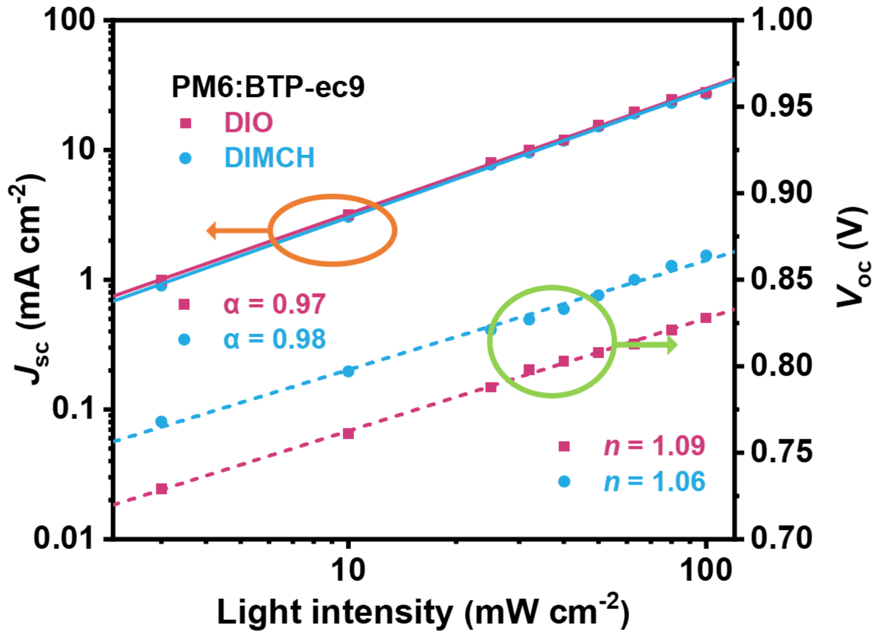

Figure S21. J_{ph} and V_{OC} versus light intensity plots of the PM6:BTP-ec9-based OSCs.

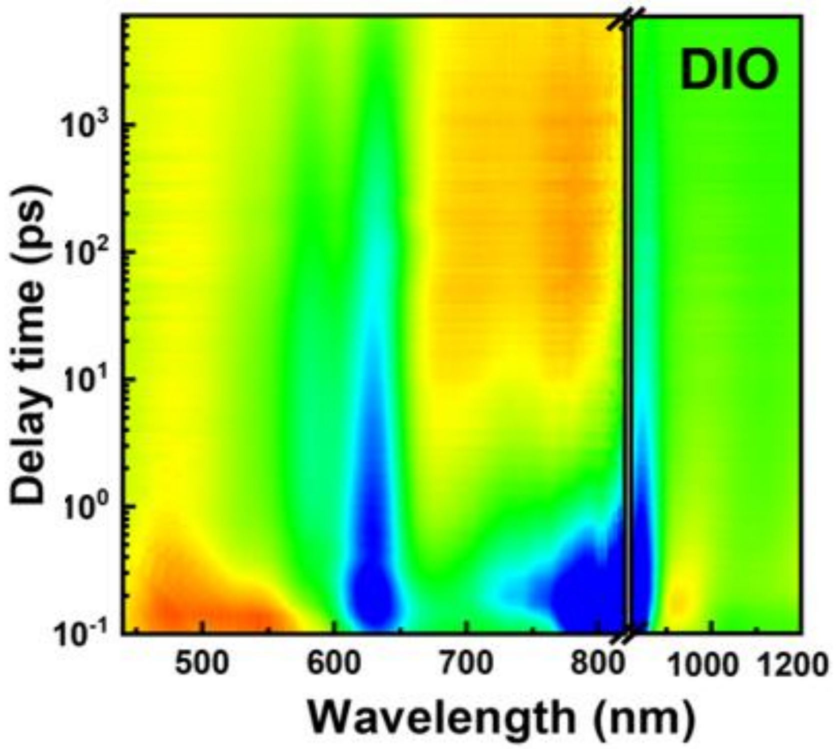

Figure S22. The 2D TA profile at different time delays of DIO treated PM6:L8-BO film pumped at 800 nm.

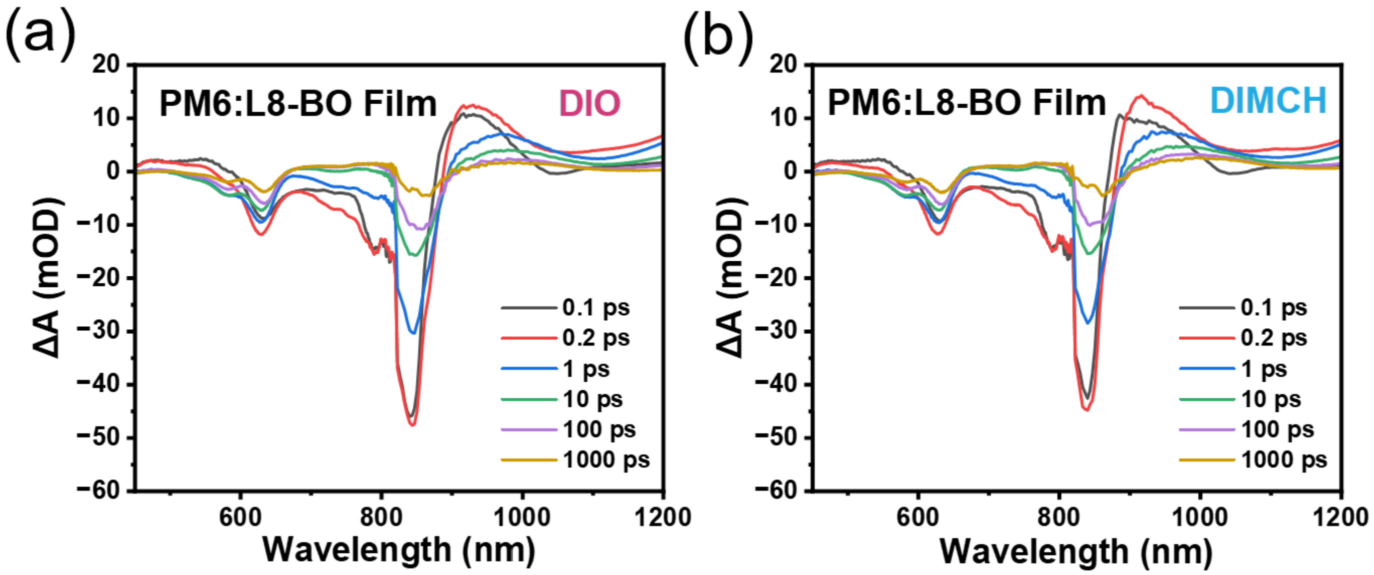

Figure S23. The TA spectra at different time delays of (a) DIO or (b) DIMCH treated PM6:L8-BO film pumped at 800 nm.

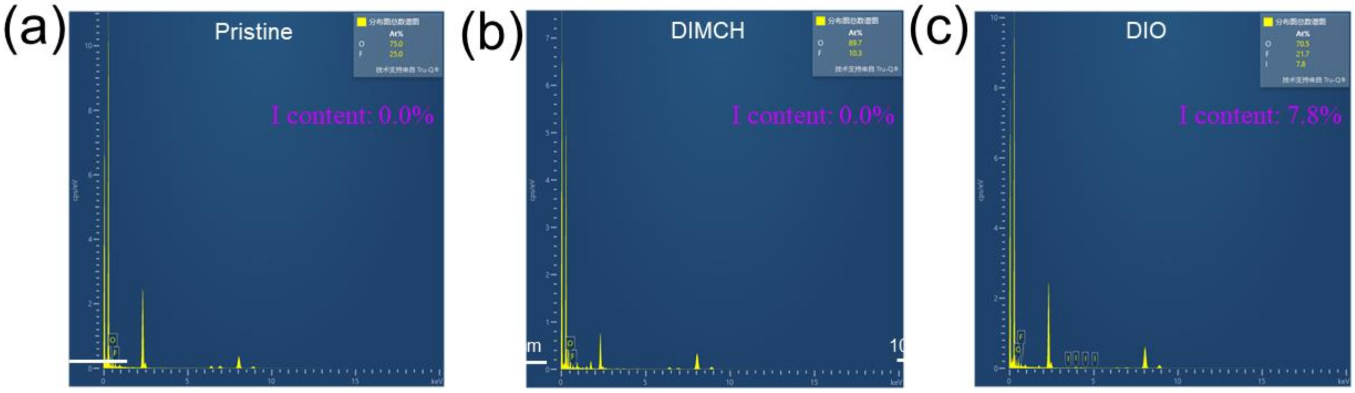

Figure S24. Transmission electron microscope-energy dispersive spectroscopy (TEM-EDS) spectra of PM6:L8-BO films with DIO or DIMCH.

Table S1. Detailed energy losses in devices based on different acceptors.

Active layer	E_g [eV]	qV_{oc} [eV]	E_{loss} [eV]	qV_{oc}^{SQ} [eV]	qV_{oc}^{rad} [eV]	ΔE_1 [eV]	ΔE_2 [eV]	ΔE_3 [eV]	EQE_{EL} [%]
PM6:Y6 (DIO)	1.410	0.821	0.589	1.145	1.059	0.265	0.086	0.238	9.97×10^{-3}

PM6:Y6 (DIMCH)	1.422	0.858	0.564	1.145	1.076	0.266	0.080	0.218	2.17×10^{-2}
PM6:BTP-ec9 (DIO)	1.404	0.828	0.576	1.140	1.053	0.264	0.086	0.226	1.57×10^{-2}
PM6:BTP-ec9 (DIMCH)	1.418	0.862	0.556	1.153	1.073	0.266	0.080	0.210	2.95×10^{-2}
PM6:L8-BO (DIO)	1.450	0.866	0.584	1.182	1.095	0.268	0.087	0.229	1.41×10^{-2}
PM6:L8-BO (DIMCH)	1.466	0.906	0.560	1.197	1.115	0.269	0.082	0.209	3.04×10^{-2}

Table S2. Morphological information revealed from the GIWAXS investigations.

Treatment	PM6, OOP direction				L8-BO, OOP direction			
	Q ($Å^{-1}$)	Distance (Å)	$FWHM_{010}$ ($Å^{-1}$)	CCL_{010} (Å)	Q ($Å^{-1}$)	Distance (Å)	$FWHM_{010}$ ($Å^{-1}$)	CCL_{010} (Å)
none	1.68	3.74	0.29	21.6	1.72	3.65	0.20	31.4
annealing	1.70	3.70	0.25	24.7				
DIO-annealing	1.71	3.67	0.24	26.9	1.76	3.61	0.17	36.9
DIMCH (15%)	1.71	3.68	0.25	25.4	1.72	3.65	0.19	33.1
DIMCH (15%)-annealing	1.73	3.63	0.21	29.2	1.73	3.63	0.20	31.4
DIMCH (50%)-annealing	1.69	3.71	0.27	23.5				

Table S3. Fitting parameters of 1D GISAXS profiles of PM6:L8-BO films processed with different additives.

Blend film	ξ (Å)	D	η (Å)	Rg (nm)
PM6:L8-BO (DIO)	32.9	2.99	83.4	23.2
PM6:L8-BO (DIMCH)	25.6	2.99	67.2	17.2

Table S4. Hole mobility values of the PM6:L8-BO-based devices at different temperatures.

Temperature (K)	DIO (cm^2 V^{-1} s^{-1})	DIMCH (cm^2 V^{-1} s^{-1})
298	2.66×10^{-4}	5.43×10^{-4}
273	2.39×10^{-4}	4.88×10^{-4}
253	1.74×10^{-4}	3.86×10^{-4}
233	1.52×10^{-4}	2.82×10^{-4}
213	9.76×10^{-5}	2.44×10^{-4}
193	4.88×10^{-5}	1.57×10^{-4}
173	2.81×10^{-5}	8.54×10^{-5}
153	1.16×10^{-5}	3.36×10^{-5}
133	3.38×10^{-6}	1.05×10^{-5}
